# AGE DIFFERENCES IN SELF-CONTINUITY REMAIN ROBUST IN RESPONSE TO THE COVID-19 PANDEMIC

**DOI:** 10.1093/geroni/igac059.1951

**Published:** 2022-12-20

**Authors:** Yi Lu, Corinna Löckenhoff

**Affiliations:** Cornell University, Ithaca, New York, United States; Cornell University, Ithaca, New York, United States

## Abstract

Self-continuity, the perceived connectedness to one’s past and future selves, predicts well-being and tends to be higher among older adults (Rutt & Löckenhoff, 2016). Self-continuity is also susceptible to major life changes (Sani, 2008). The pandemic brought dramatic changes in multiple life domains, especially for older adults who experienced higher rates of COVID-19 morbidity and mortality and may have been more likely to restrict their behavior as a result. This study examined whether age differences in self-continuity remained robust in response to the pandemic using representative U.S. survey data. Pre-pandemic data (Fall 2016, N = 230, aged 18 – 87, M = 50.85, SD = 16.40, 47.83% female, 67.83% white) and mid-pandemic data (Summer 2020, N = 230, aged 18 – 88, M = 50.20, SD = 19.49, 48.27% female, 64.78% white) were demographically matched using propensity scores. Participants rated their self-continuity one, five, and ten years into the past and future. Multi-level modeling examined the effects of age, temporal distance (in years), temporal direction (past vs. future), and assessment time (pre- vs. mid-pandemic) on self-continuity. Consistent with prior research, self-continuity was lower for more distant intervals, especially for the past. An interaction between temporal direction and assessment time indicated that future self-continuity was lower mid-pandemic than pre-pandemic. Across samples, however, advanced age was associated with higher self-continuity and the size of this effect did not vary by assessment time. Overall, findings suggest that even though future self-continuity decreased during the pandemic, existing age effects remained robust to this disruption.

